# Artificial intelligence-driven exercise programmes in personalising the management of multimorbidity

**DOI:** 10.3399/BJGPO.2025.0094

**Published:** 2025-09-24

**Authors:** Jacob Keast, Glenn Simpson, Lucy Smith, Hajira Dambha-Miller

**Affiliations:** 1 University of Exeter Medical School, Exeter, UK; 2 Primary Care Research Centre, University of Southampton, Southampton, UK

**Keywords:** artificial intelligence, exercise programme, multimorbidity, general practitioners, primary healthcare

Multimorbidity, the presence of two or more chronic conditions, presents significant challenges in health care. Multimorbidity affects over 25% of UK adults and is a growing global challenge, contributing substantially to disability-adjusted life years (DALYs) and healthcare costs.^1^ Long-term conditions, which frequently co-occur, now account for over 70% of global DALYs, underscoring the urgency of scalable, cost-effective interventions.^2^ Individuals with multimorbidity often struggle with complex treatment regimens, multiple medications, and care plans tailored to each condition, leading to fragmented care that may not address their overall health needs.^3^ Exercise is an important component in the management of chronic conditions, although there is a significant challenge in designing personalised approaches to accommodate the unique combination of health conditions a patient faces. Limitations of standardised exercise referral schemes include limited adaptability to individual progress, low adherence rates, and a lack of contextual personalisation^4,5^ Artificial intelligence (AI)-driven exercise programmes offer a promising solution, providing tailored and adaptable plans that respond to the specific needs of populations affected by multimorbidity. AI coaching models have a well-established presence in the sports industry; examples include TrainerRoad and Tri-Dot, which optimise training plans with feedback and review from each session’s outcomes.^6^ These models, developed over a decade, offer high quality, data-driven solutions that improve performance and engagement. For chronic health conditions, AI can similarly optimise exercise plans based on patients’ needs. For example, individuals with diabetes benefit from a mixed training approach with a preference for high-intensity efforts that stimulate hypertrophy and improve muscle glucose uptake, enhancing insulin sensitivity. Exercise has also been shown to improve pulmonary function and exercise tolerance in individuals with chronic obstructive pulmonary disease (COPD).^7^ In people with osteoarthritis and type 2 diabetes, structured aerobic and resistance training is associated with reduced joint stiffness and significant decreases in HbA₁c levels.^8–10^ This personalisation represents a significant advancement in exercise for chronic disease management compared with current models.

Additionally, in managing chronic diseases, exercise plays a central role in the improvement of cardiovascular health, reducing blood glucose levels, strengthening muscles, and alleviating joint pain.^11^ However, to achieve this, the regimen for individuals with multimorbidity must be tailored to their unique health profile. A one-size-fits-all approach may not always be appropriate, as each condition requires distinct considerations. For example, a patient with both diabetes and osteoarthritis requires an exercise programme addressing cardiovascular health and joint pain relief. Where the aforementioned high-intensity exercise is beneficial in diabetes, a lower impact or more targeted programme may be necessary for osteoarthritis. Further, an exercise programme may also account for a patient’s personal characteristics and their wider environment, such as age, sex, socioeconomic circumstances, and other factors . AI-driven exercise programmes can optimise these plans by leveraging data to create safe, effective regimens suited to each condition and to an individual’s personal context. AI’s ability to dynamically adapt exercise programmes is a key feature that sets it apart from traditional approaches. AI-driven exercise platforms can dynamically adjust activity recommendations based on real-time physiological feedback, for example, modifying training intensity in response to heart rate variability deviations from baseline or adapting exercise load based on wearables-driven VO₂ max estimates.^12,13^ Conventional exercise plans are static, unable to respond to changes in a patient’s health state or fitness level. AI systems, however, monitor key health and exercise metrics, adjusting intensity based on patient feedback and tracked data. For example, if a patient over-exerts themselves one day, a plan can reduce subsequent session intensity to allow for improved recovery and long-term benefits. Over time, exercise plans can ensure continuous adaptation and health improvements, enabling safe, effective, and evolving exercise regimens.^14,15^

Adherence to physical activity is a significant challenge for individuals with chronic conditions. By personalising the intensity and type of exercise, AI-driven programmes make physical activity more accessible and engaging. This approach, combined with real-time feedback and progress tracking, may increase the likelihood that individuals continue with their exercise plans.^16^ Wearable devices integrated with AI platforms provide timely updates on progress, celebrate milestones, and issue reminders, thus maintaining patient motivation. This level of engagement is especially important for populations with multimorbidity, who may require additional support. While AI can deliver personalised exercise programmes, human interaction remains essential for accountability and engagement. Health coaches play pivotal roles in supporting patients by monitoring progress, providing guidance, and ensuring adherence to the programme.^17^ The combination of AI-generated plans and human oversight promotes sustained engagement and improves health outcomes, with potential for improved service capacity. Features such as check-ins, messaging, goal setting, and peer-to-peer support functions help maintain adherence and foster a sense of community among individuals with similar health challenges.^17^

AI-powered exercise programmes also offer significant benefits in terms of accessibility and scalability. For instance, tailored cardiac rehabilitation programmes could increase the breadth of conditions included for rehabilitation, as well as aiding those in rural areas who struggle to access in-person services. AI-powered interventions provide these patients with the support they need to engage in exercise, improving health outcomes. These systems, available via web apps and other devices, offer a flexible and accessible alternative for individuals unable to attend rehabilitation sessions due to mobility or travel constraints. From an operational standpoint, AI-driven exercise programmes can address capacity issues within healthcare systems, for example, long waiting lists for Tier 3 weight management services in the UK create a backlog of patients.^18^ AI exercise prescription software can provide an initial low-level intervention to improve service delivery without overwhelming healthcare systems. Moreover, it can bridge the gap in specialist expertise by enabling general health coaches, such as Level 2 fitness instructors, to facilitate exercise plans, reducing the reliance on specialist trainers.

The potential of AI-driven exercise programmes extends beyond physical health to address mental health. Individuals with multimorbidity often experience anxiety, depression, and other mental health challenges. These systems can integrate mental health support within exercise programmes, recommending stress-reducing activities such as yoga or mindfulness exercises alongside more physically demanding tasks. This holistic approach fosters both physical and emotional wellbeing, improving overall chronic disease management.

In the future, AI-driven exercise programmes could offer revolutionary chronic condition management by providing truly personalised and scalable solutions. Iterating algorithms over time can increase plan resolution, or scope, by adapting to additional health conditions and medications, expanding their applicability across clinical areas within the NHS. For example, the NHS GP Exercise Referral Scheme currently advises on activity for 13 long-term health conditions, but similar exercise principles can be developed and applied for cardiac rehabilitation, fitness for surgery, pulmonary rehabilitation, obesity clinics, falls prevention, and more. AI-informed exercise platforms, such as VITOVA, Sweatcoin**,** EXi**,** MyRecovery**,** Kaido**,** Changing Health**,** and AMP (Advanced Movement Programming), use various algorithms to personalise exercise plans based on health conditions, or medications, and/or patient-derived goals.^10^ Future iterations incorporating generative AI and machine learning models can further optimise these plans, enhancing and informing chronic disease management.

Through continuous monitoring, real-time adjustments, and bespoke support, these systems address key challenges in chronic disease management, ultimately improving health, quality of life, and long-term prognoses. The clinical implications are substantial: AI and exercise prescription software have the potential to revolutionise exercise support through scalable, cost-effective, and personalised interventions that can be adapted for diverse patient populations, yielding profound improvements in chronic disease management. While some GP practices have begun to adopt these platforms, large-scale evaluations are essential to confirm their efficacy and optimise software integration within healthcare systems. Moreover, NHS collaboration with commercial industry partners in AI-informed physical activity will be critical to rapidly scale and implement these technologies, ensuring broad accessibility and maximising the positive impacts on chronic health condition management going forward. Finally, to support implementation at scale, future integration of AI-enabled platforms into NHS pathways should be accompanied by economic modelling, including cost-effectiveness analyses and return-on-investment projections, to strengthen the case for sustainable adoption.

## Data

N/A
